# STATE USE OF VALUE-BASED PAYMENT IN NURSING FACILITIES

**DOI:** 10.1093/geroni/igac059.1315

**Published:** 2022-12-20

**Authors:** Denise Tyler, Kristie Porter, Melissa Hunter, Iara Oliveira, Marie Squillace, Judith Dey

**Affiliations:** RTI International, Waltham, Massachusetts, United States; RTI International, Durham, North Carolina, United States; RTI International, Durham, North Carolina, United States; Office of the Assistant Secretary for Planning and Evaluation, Washington, District of Columbia, United States; ASPE, Washington, District of Columbia, United States; Office of the Assistant Secretary for Planning and Evaluation, Washington, District of Columbia, United States

## Abstract

Some states use value-based payment (VBP) programs as part of their nursing facility Medicaid payment systems. The purpose of this study was to determine which states use these programs, their goals, the measures used and other elements of these programs. An environmental scan was conducted to identify states that have VBP programs for nursing facilities, the key design elements of those programs, and the goals that states are aiming to achieve. Interviews with seven stakeholders were also conducted. We found that 20 states and the District of Columbia use VBP for nursing facilities with the goals of improving quality and increasing efficiency. However, due to lack of evaluation, it is not known if these programs are achieving their goals. Additional research is needed to determine which state programs are most successful. The results of such research would help in the development of future VBP programs.

